# National Trends in Atrial Fibrillation Hospitalizations, Readmissions, and Mortality, 2010 to 2022

**DOI:** 10.1016/j.jacadv.2026.103118

**Published:** 2026-08-12

**Authors:** Abdulhaseeb Khan, Manyoo A. Agarwal, Peter Hanna, Boback Ziaeian

**Affiliations:** a Department of Internal Medicine, University of California-Los Angeles, Los Angeles, California, USA; b Division of Cardiovascular Medicine, Heart Vascular Thoracic Institute, Cleveland Clinic Abu Dhabi, Abu Dhabi, UAE; c Division of Cardiology, University of California-Los Angeles, Los Angeles, California, USA

**Keywords:** atrial fibrillation, readmissions, in-hospital mortality, National Readmission Database, rhythm management

## Abstract

**Background:**

Atrial fibrillation (AF) is a leading cause for acute cardiovascular hospitalizations. There is limited contemporary data about national trends and risk factors that predict readmission and mortality.

**Objectives:**

The objective of the study was to identify national trends in hospitalizations, readmissions, and inpatient mortality during index admission for AF.

**Methods:**

Using nationally representative, survey-weighted hospitalization data, we assessed AF hospitalization trends and calculated risk factors for 30-day readmission and in-hospital mortality using cluster-adjusted multivariable logistic regression.

**Results:**

Between 2010 and 2022, there were 6,141,046 primary AF hospitalizations among 5,528,397 unique patients, of which 892,254 (16.1%) experienced 30-day readmission and 53,084 (0.96%) deaths during index admission. Renal disease was the strongest predictor of both 30-day readmission (adjusted OR: 1.39; 95% CI: 1.37-1.40) and in-hospital mortality (adjusted OR: 3.98; 95% CI: 3.85-4.11); public insurance was also strongly associated with adverse outcomes, including Medicare (readmission OR: 1.46; mortality OR: 1.17) and Medicaid coverage (readmission OR: 1.62; mortality OR: 1.43), along with lower income quartile, chronic obstructive pulmonary disease (readmission OR: 1.48; mortality OR: 1.66), heart failure (readmission OR: 1.38; mortality OR: 1.83), prior myocardial infarction (readmission OR: 1.26; mortality OR: 3.34), and female sex (readmission OR: 1.12; mortality OR: 1.07). Total cost of AF hospitalizations in 2022 was $7.2 billion.

**Conclusions:**

In the United States, AF hospitalizations and readmissions experienced a modest overall net increase from 2010 to 2022, with a notable decline from 2014 to 2020 before rebounding following the COVID-19 pandemic, and 30-day readmissions and in-hospital mortality were associated with age, comorbidities, and socioeconomic factors.

Atrial fibrillation (AF) is the most common heart rhythm disorder and is estimated to affect 10.5 million individuals in the United States.^1^ As a result, symptomatic AF is a leading cause of cardiovascular (CV) hospitalizations and readmissions in the United States.^2^ An estimated $28.4 billion was spent nationally in direct health care spending for AF in 2016, corresponding to approximately $36 billion when inflation-adjusted, with hospitalizations and readmissions being the primary cost drivers and costs rising over the past decade.^3^^,^^4^

Although prior analyses have examined readmissions following AF hospitalization, these studies predate the widespread adoption of direct oral anticoagulants and contemporary rhythm control strategies.^3^^,^^5^ Furthermore, they are limited by short-term, cross-sectional designs confined to single-year analyses and therefore do not capture longitudinal trends.^6^ Accordingly, contemporary national data examining long-term hospitalization patterns and outcomes are needed. We examined contemporary trends of unique primary AF hospitalization and factors associated with 30-day readmission visits as well as in-hospital mortality on index admission in a large national U.S. administrative database from 2010 to 2022.

## Methods

We estimated national rates of hospitalization and readmission for Americans admitted with AF using the Nationwide Readmissions Database (NRD) as performed previously.^7^, ^8^, ^9^, ^10^, ^11^ The NRD is a publicly available all-payer database developed by the Agency for Healthcare Research and Quality for the Healthcare Cost and Utilization Project. The NRD provides accurate representation of total U.S. hospitalizations and readmissions visits regardless of insurance provider. We identified all adult (18 years or older) hospitalizations from January 1, 2010, to November 30, 2022, with an AF discharge diagnosis in the primary or secondary diagnosis fields. AF was defined by International Classification of Diseases- 9th Revision-Clinical Modification (ICD-9-CM) and ICD-10-CM codes (ICD-10-CM codes I48.1 to I48.91; ICD-9-CM codes 427.31); atrial flutter diagnoses (ICD-9-CM 427.32 and ICD-10-CM I48.92) were excluded. A primary AF hospitalization was defined as any ICD-9-CM or ICD-10-CM AF discharge code used as the primary designated diagnostic code. The NRD uses unique identifiers for patients to account for readmissions within and limited to a given year. The NRD does not allow tracking of patients across calendar years; index hospitalizations discharged in December were excluded to ensure complete 30-day follow-up for readmission outcomes. The NRD captures only formal inpatient admissions and does not include observation-status stays.

We examined calendar-year changes in primary AF hospitalizations, including overall and unique patient visits (those with 1 visit only vs those with readmissions). The patient demographics (age, insurance, and median household income national quartiles) and comorbidities (obesity, heart failure [HF], history of myocardial infarction [MI], hypertension [HTN], diabetes mellitus, hyperlipidemia [HLD], coronary artery disease [CAD], cerebrovascular disease, chronic obstructive pulmonary disease [COPD], and renal disease) were described for the overall cohort with a primary diagnosis of AF and were assessed by regressing each characteristic on calendar year, modeled as a continuous variable. Linear regression was used for continuous variables and logistic regression for categorical variables, with p-trend reported. Calendar year was modeled as a continuous variable to characterize directional trends in patient demographics and comorbidities over the study period, rather than to identify specific inflection points in trend direction. The NRD does not include race/ethnicity data for hospitalizations.

Baseline characteristics of patients based on readmission status and index hospitalization mortality were compared using survey-weighted means with SEs for continuous variables and survey-weighted proportions for categorical variables, with differences assessed using linearized Wald tests and Rao-Scott chi-square tests, respectively. Standardized mean differences were calculated to quantify the magnitude of between-group differences in baseline characteristics. Survey-weighted analyses accounted for the NRD's complex sampling design, with discharge weights applied as probability weights, stratification by NRD stratum, and clustering at the hospital level to account for within-hospital correlation of outcomes. Survey-weighted multivariable logistic regression was used to calculate the OR with 95% CI for 30-day readmission as well as death on index admission with adjustment for baseline demographic characteristics and comorbidities selected a priori based on clinical plausibility and prior established associations with AF outcomes, including age, sex, payer status, income quartile, obesity, HF, history of MI, HTN, diabetes mellitus, HLD, CAD, cerebrovascular disease, COPD, and renal disease.^5^^,^^12^^,^^13^ All analyses were performed in Stata (version 19.5; StataCorp). The University of California-Los Angeles Institutional Review Board deemed this study exempt from review.

## Results

**Figure 1:**
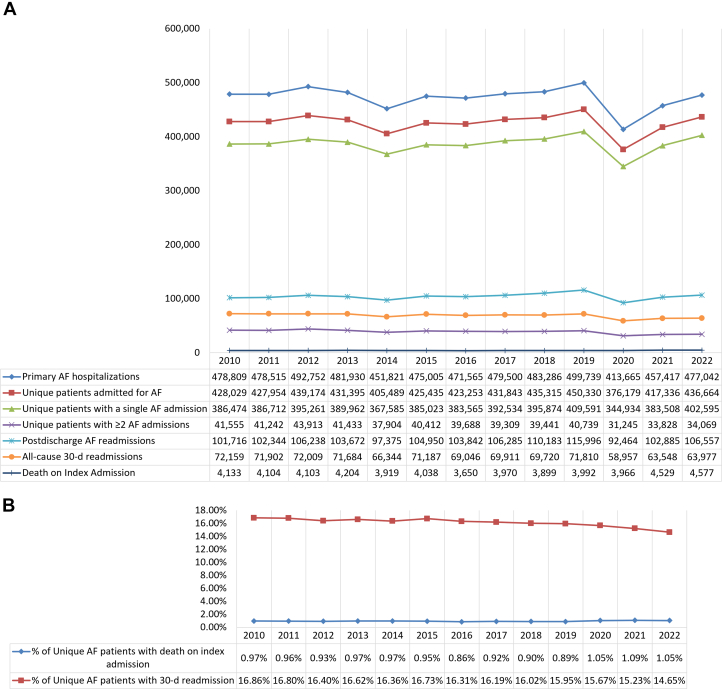
Trends for Atrial Fibrillation Hospitalizations, Unique Patients, and Readmissions (A) Trends in admissions, 30-d readmissions, and death on index admission for AF. (B) Percent of unique AF patients with death on index admission and 30-d readmission. AF = atrial fibrillation.

Between 2010 and 2022 in the United States, there were 56,104,334 (unweighted, 28,321,118) hospitalizations with a primary or secondary diagnosis of AF and 6,141,046 primary AF hospitalizations. There were 5,528,396 unique patients, of which 5,023,618 patients experienced a single primary AF admission, whereas 504,778 patients had 2 or more primary AF hospitalizations within 1 calendar year (Figure 1). There were 1,354,507 postdischarge AF readmissions identified across the study period, of which 892,254 represented first all-cause readmissions occurring within 30 days of a primary AF index hospitalization and constituted the primary readmission outcome for this analysis. After applying hospital-specific cost-to-charge ratios and diagnosis-related group–based case-mix adjustment, the total estimated cost of primary AF hospitalizations was $7.2 billion in 2022.

**Figure 2:**
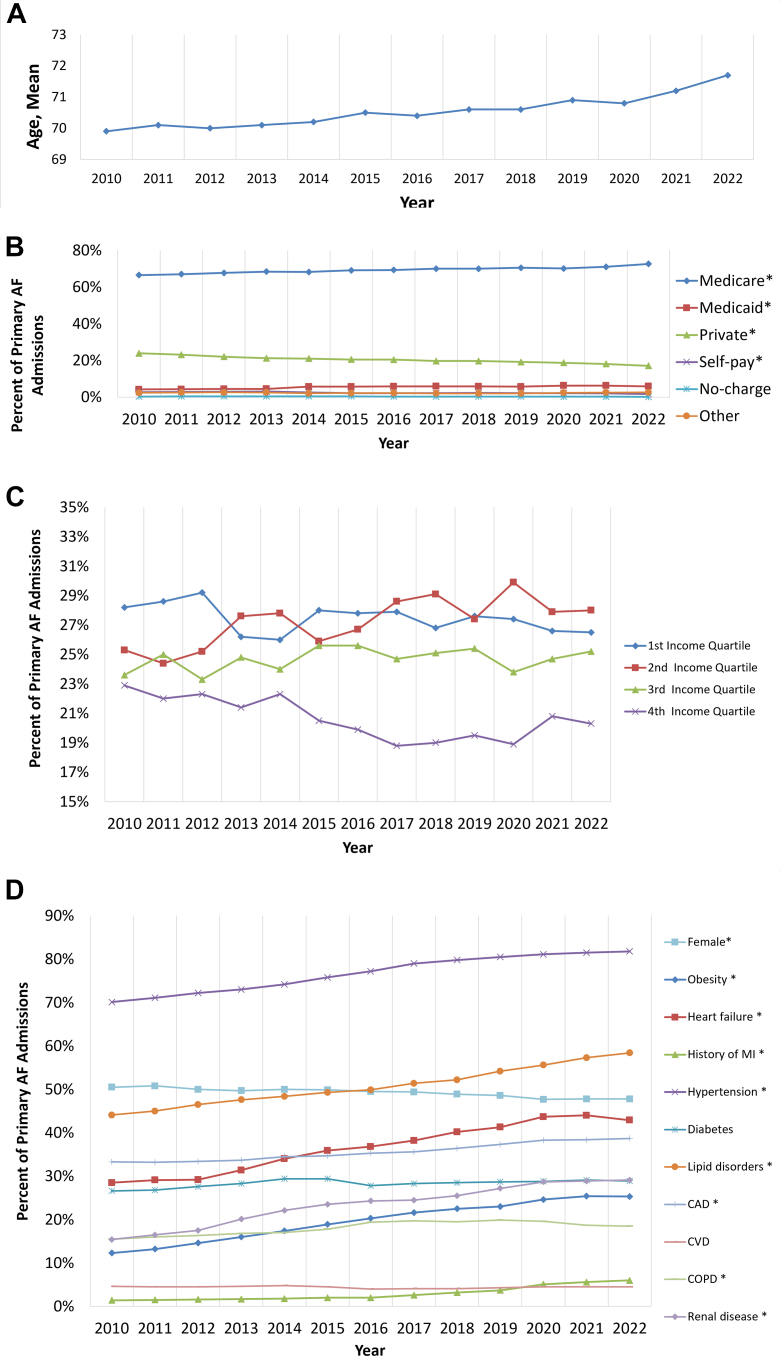
Trends in Demographics and Comorbidities for Primary AF Hospitalization (A) Mean age of primary AF patients from 2010 to 2022, (B) percent of primary AF admissions by payer type, (C) percent of primary AF admissions by income quartile, 1st quartile represents highest income quartile, (D) percent of primary AF admissions with corresponding comorbidity ∗Indicates *P*-trend <0.001. CAD = coronary artery disease; CVD = cerebrovascular disease; COPD = chronic obstructive pulmonary disease; MI = myocardial infarction; other abbreviation as in Figure 1.

The mean age at index hospitalization was 70.5 (95% CI: 70.5-70.6) years, and 49.3% (95% CI: 49.2-49.4) were females. The mean (SD) age of patients increased from 69.9 (13.9) years in 2010 to 71.8 (12.2) years in 2022 (*P*-trend <0.001), whereas the proportion of females decreased (49.5% [95% CI: 49.1-49.9] in 2010 to 47.8% [95% CI: 47.4-48.1] in 2022; *P*-trend <0.001). The proportion of patients covered by Medicare increased to 72.6% (*P*-trend <0.001), whereas private insurance and self-pay hospitalizations declined to 17.1% (*P*-trend <0.001) and 1.6% (*P*-trend = 0.002), respectively. The first- and second-income quartiles accounted for most patients across the study period, with 26.5% and 28.0% in 2022, respectively (*P*-trend = 0.10 and 0.04). Overall, the prevalence of comorbid conditions rose substantially, with notable increases in obesity (12.3% to 25.3%; *P*-trend <0.001), HF (28.5% to 42.9%; *P*-trend <0.001), renal disease (15.4% to 29.1%; *P*-trend <0.001), and lipid disorders (44.1% to 58.4%; *P*-trend <0.001). HTN remained the most prevalent comorbidity, affecting 81.8% of hospitalized patients by 2022 (*P*-trend = 0.08) (Figure 2, Central Illustration, Supplemental Table 3).

**Central Illustration:**
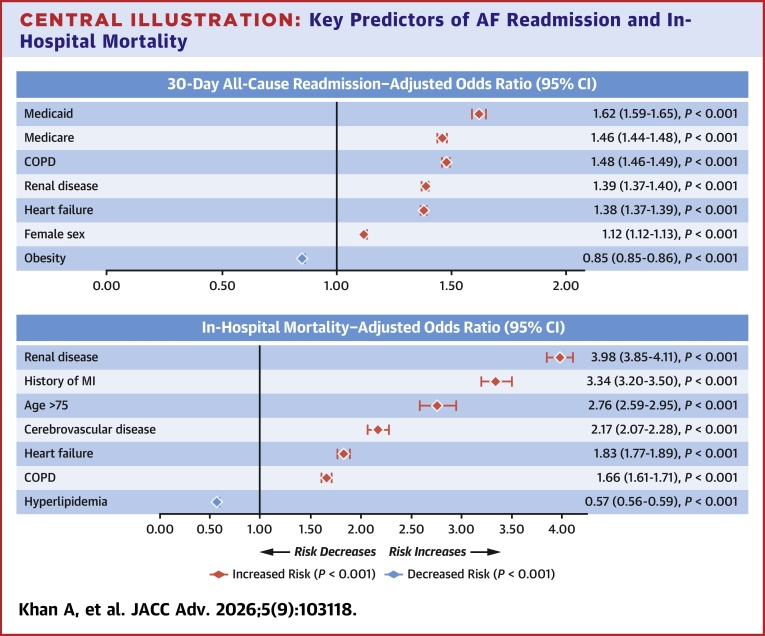
Key Predictors of AF Readmission and In-Hospital Mortality Using nationally representative data from the Nationwide Readmissions Database (2010-2022), renal disease, heart failure, COPD, and public insurance coverage were the strongest predictors of 30-day readmission, while renal disease, history of myocardial infarction, age >75, and cerebrovascular disease were most strongly associated with in-hospital mortality following primary AF hospitalization, highlighting the disproportionate burden of comorbidity and socioeconomic disadvantage on short-term outcomes. AF = atrial fibrillation; COPD = chronic obstructive pulmonary disease; MI = myocardial infarction.

There were 892,254 30-day readmissions after primary AF hospitalization, which represented 16.1% of the index population. Readmitted patients were older (72.7 vs 70.3 years) and more frequently covered by Medicare (76.7% vs 32.1%) or Medicaid (5.9% vs 4.9%), with lower representation of private insurance and self-pay (*P* < 0.001). Comorbidities were more prevalent among readmitted patients, with HTN affecting 79.3%, followed by HF (45.9%), CAD (42.7%), diabetes (33.0%), and renal disease (31.8%) (Table 1). Female sex (1.12, 1.12-1.13), Medicare (1.46, 1.44-1.48), Medicaid (1.62, 1.59-1.65), HF (1.38, 1.37-1.39), history of MI (1.26, 1.23-1.28), COPD (1.48, 1.46-1.49), and renal disease (1.39, 1.37-1.40) were associated with higher odds of 30-day readmission. Patients in the highest income quartile had lower adjusted odds of 30-day readmission (OR: 0.94; 95% CI: 0.92-0.95) compared to the lowest income quartile (Table 2).

**Table 1:** Baseline Characteristics Comparing Patients Readmitted vs Not Readmitted Within 30 Days

	Overall (N = 5,528,397)	Not Readmitted Within 30 Days (n = 4,636,143)	Readmitted Within 30 Days (n = 892,254)	Standardized Mean Difference	*P* Value
Age, y, mean (SE)	70.7 (0.03)	70.3 (0.03)	72.7 (0.03)	0.1840	<0.001
Female	2,858,129 (51.7%)	2,393,782 (51.6%)	464,347 (52.0%)	0.0712	<0.001
Payer status					
Medicare	2,171,640 (39.3%)	1,487,559 (32.1%)	684,081 (76.7%)	0.1883	<0.001
Medicaid	280,628 (5.1%)	227,983 (4.9%)	52,645 (5.9%)	0.0414	<0.001
Private insurance	1,134,298 (20.5%)	1,013,785 (21.9%)	120,513 (13.5%)	−0.2156	<0.001
Self-pay	128,871 (2.3%)	114,373 (2.5%)	14,498 (1.6%)	−0.0549	<0.001
No charge	16,368 (0.3%)	14,357 (0.3%)	2,011 (0.2%)	−0.0135	<0.001
Other payer	127,013 (2.3%)	109,780 (2.4%)	17,233 (1.9%)	−0.0286	<0.001
Median household income quartile					
1st	1,482,565 (26.8%)	1,225,126 (26.4%)	257,439 (28.9%)	0.0538	<0.001
2nd	1,484,014 (26.8%)	1,242,974 (26.8%)	241,040 (27.0%)	0.066	0.023
3rd	1,350,092 (24.4%)	1,137,900 (24.5%)	212,192 (23.8%)	−0.0180	<0.001
4th	1,136,659 (20.6%)	966,640 (20.8%)	170,019 (19.1%)	−0.0438	<0.001
Comorbidities					
Hypertension	4,228,947 (76.5%)	3,521,038 (75.9%)	707,909 (79.3%)	0.0847	<0.001
Diabetes	1,556,313 (28.2%)	1,261,523 (27.2%)	294,790 (33.0%)	0.1288	<0.001
Lipid disorders	2,801,250 (50.7%)	2,342,727 (50.5%)	458,523 (51.4%)	0.0184	<0.001
Obesity	1,072,518 (19.4%)	914,248 (19.7%)	158,270 (17.7%)	−0.0486	<0.001
Heart failure	1,967,926 (35.6%)	1,557,959 (33.6%)	409,967 (45.9%)	0.2558	<0.001
CAD	1,952,814 (35.3%)	1,572,202 (33.9%)	380,612 (42.7%)	0.1794	<0.001
History of MI	166,944 (3.0%)	128,902 (2.8%)	38,042 (4.3%)	0.0799	<0.001
CVD	249,801 (4.5%)	198,898 (4.3%)	50,903 (5.7%)	0.0650	<0.001
COPD	975,943 (17.6%)	752,656 (16.2%)	223,287 (25.0%)	0.2186	<0.001
Renal disease	1,280,435 (23.2%)	996,699 (21.5%)	283,736 (31.8%)	0.2358	<0.001

CAD = coronary artery disease; COPD = chronic obstructive pulmonary disease; CVD = cerebrovascular disease; MI = myocardial infarction.

**Table 2:** Multivariable-Adjusted Predictors of 30-Day All-Cause Readmission Following Primary Atrial Fibrillation Hospitalization

	Adjusted OR (95% CI)	*P* Value
Age, y		
<65	Reference	Reference
65–75	0.94 (0.92-0.95)	<0.001
>75	1.01 (0.99-1.02)	0.246
Sex		
Male	Reference	Reference
Female	1.12 (1.12-1.13)	<0.001
Payer Status		
Private	Reference	Reference
Medicare	1.46 (1.44-1.48)	<0.001
Medicaid	1.62 (1.59-1.65)	<0.001
Self-pay	1.00 (0.97-1.03)	0.957
No charge	1.11 (1.01-1.21)	0.023
Other	1.16 (1.12-1.19)	<0.001
Median income quartile		
1st	Reference	Reference
2nd	0.96 (0.95-0.97)	<0.001
3rd	0.95 (0.94-0.96)	<0.001
4th	0.94 (0.92-0.95)	<0.001
Comorbidities		
Obesity	0.85 (0.85-0.86)	<0.001
Heart failure	1.38 (1.37-1.39)	<0.001
History of MI	1.26 (1.23-1.28)	<0.001
Hypertension	1.01 (1.00-1.02)	0.279
Diabetes mellitus	1.19 (1.18-1.20)	<0.001
Hyperlipidemia	0.91 (0.91-0.92)	<0.001
Coronary artery disease	1.23 (1.22-1.24)	<0.001
Cerebrovascular disease	1.18 (1.16-1.20)	<0.001
Chronic obstructive pulmonary disease	1.48 (1.46-1.49)	<0.001
Renal disease	1.39 (1.37-1.40)	<0.001

Abbreviation as in Table 1.

During index admission, 53,084 deaths occurred representing 0.96% of admissions (Table 3). Similar to 30-day readmissions, patients who died were older (78.1 vs 70.6 years), more frequently females (54.6% vs 49.0%), and more often covered by Medicare (84.1% vs 69.2%). Comorbidities were also more prevalent in this population, most commonly HF (60.4%), renal disease (59.7%), history of MI (12.9%), COPD (30.9%), and CAD (40.0%) (Table 3). In multivariable analysis, higher odds of in-hospital death were observed with older age, with patients aged 65 to 75 (1.60, 1.50-1.71) and those >75 (2.76, 2.59-2.95). In addition, female sex (1.07, 1.04-1.10), Medicare (1.17, 1.09-1.25) and Medicaid (1.43, 1.32-1.56) coverage, and comorbidities including HF (1.83, 1.77-1.89), history of MI (3.34, 3.20-3.50), cerebrovascular disease (2.17, 2.07-2.28), COPD (1.66, 1.61-1.71), and renal disease (3.98, 3.85-4.11) were associated with an increased risk of mortality on index admission. Similar to readmissions, obesity (0.66, 0.63-0.69) and HLD (0.57, 0.56-0.59) were associated with lower odds of death. Patients in the highest income quartile had lower adjusted odds of in-hospital mortality (OR: 0.80; 95% CI: 0.77-0.84) compared to the lowest income quartile (Table 4).

**Table 3:** Baseline Characteristics Comparing Patients Died vs Survived Index Hospitalization

	Overall (N = 5,526,772)	Survived (n = 5,473,688)	Died (n = 53,084)	Standardized Mean Difference	*P* Value^a^
Age, y, mean (SE)	70.7 (0.03)	70.6 (0.03)	78.1 (0.08)	0.5962	<0.001
Female	2,708,118 (49.0%)	2,682,107 (49.0%)	26,011 (54.6%)	0.1086	<0.001
Payer status					
Medicare	3,829,053 (69.3%)	3,788,008 (69.2%)	41,045 (84.1%)	0.3473	<0.001
Medicaid	282,865 (5.1%)	278,158 (5.1%)	2,077 (3.9%)	−0.0623	<0.001
Private insurance	1,133,993 (20.5%)	1,127,576 (20.6%)	4,298 (8.1%)	−0.3494	<0.001
Self-pay	127,116 (2.3%)	125,895 (2.3%)	637 (1.2%)	−0.0866	<0.001
No charge	16,580 (0.3%)	16,421 (0.3%)	53 (0.1%)	−0.0425	<0.001
Other	136,165 (2.3%)	132,631 (2.3%)	1,274 (2.4%)	0.0060	0.330
Median income quartile					
1st	1,482,181 (26.8%)	1,467,911 (26.8%)	16,496 (31.1%)	0.0874	<0.001
2nd	1,482,181 (26.8%)	1,467,911 (26.8%)	14,476 (27.3%)	0.0083	0.133
3rd	1,348,540 (24.4%)	1,336,579 (24.4%)	12,046 (22.7%)	−0.0336	<0.001
4th	1,215,869 (22.0%)	1,201,287 (22.0%)	9,073 (17.6%)	−0.0689	<0.001
Comorbidities					
Obesity	1,072,192 (19.4%)	1,067,351 (19.5%)	5,893 (11.1%)	−0.2307	<0.001
Heart failure	1,968,486 (35.6%)	1,933,211 (35.3%)	32,089 (60.4%)	0.5183	<0.001
History of MI	165,803 (3.0%)	158,737 (2.9%)	6,850 (12.9%)	0.3798	<0.001
Hypertension	4,229,832 (76.5%)	4,188,671 (76.5%)	38,621 (72.5%)	−0.0871	<0.001
Diabetes mellitus	1,558,554 (28.2%)	1,543,593 (28.2%)	15,654 (29.5%)	0.0260	<0.001
Hyperlipidemia	2,803,071 (50.7%)	2,792,580 (51.0%)	20,704 (39.0%)	−0.2371	<0.001
Coronary artery disease	1,951,936 (35.3%)	1,934,183 (35.3%)	21,234 (40.0%)	0.0962	<0.001
Cerebrovascular disease	248,705 (4.5%)	246,316 (4.5%)	5,792 (10.9%)	0.2397	<0.001
COPD	973,707 (17.6%)	957,864 (17.5%)	16,412 (30.9%)	0.3124	<0.001
Renal disease	1,282,214 (23.2%)	1,258,948 (23.0%)	31,456 (59.7%)	0.8104	<0.001

Abbreviations as in Table 1.

a Died vs survived.

**Table 4:** Multivariable-Adjusted Predictors of In-Hospital Mortality During Index Admission for Primary Atrial Fibrillation Hospitalization

	Adjusted OR (95% CI)	*P* Value
Age, y		
<65	Reference	Reference
65–75	1.60 (1.50-1.71)	<0.001
>75	2.76 (2.59-2.95)	<0.001
Sex		
Male	Reference	Reference
Female	1.07 (1.04-1.10)	<0.001
Payer status		
Private	Reference	Reference
Medicare	1.17 (1.09-1.25)	<0.001
Medicaid	1.43 (1.32-1.56)	<0.001
Self-pay	1.15 (1.00-1.33)	0.051
No charge	0.75 (0.51-1.11)	0.154
Other	1.61 (1.44-1.80)	<0.001
Median income quartile		
1st	Reference	Reference
2nd	0.89 (0.86-0.92)	<0.001
3rd	0.84 (0.80-0.87)	<0.001
4th	0.80 (0.77-0.84)	<0.001
Comorbidities		
Obesity	0.66 (0.63-0.69)	<0.001
Heart failure	1.83 (1.77-1.89)	<0.001
History of MI	3.34 (3.20-3.50)	<0.001
Hypertension	0.57 (0.55-0.59)	<0.001
Diabetes mellitus	0.98 (0.95-1.01)	0.193
Hyperlipidemia	0.57 (0.56-0.59)	<0.001
Coronary artery disease	0.89 (0.86-0.91)	<0.001
Cerebrovascular disease	2.17 (2.07-2.28)	<0.001
Chronic obstructive pulmonary disease	1.66 (1.61-1.71)	<0.001
Renal disease	3.98 (3.85-4.11)	<0.001

Abbreviation as in Table 1.

## Discussion

In this nationally representative sample of hospital admissions in the United States, we found that primary AF hospitalizations declined from 2014 to 2020, rebounded in 2021 to 2022, with a modest net increase over the full study period. Thirty-day readmissions followed a similar pattern.

The large drop in admissions observed in 2020 parallels similar reports that admissions fell during the COVID-19 pandemic in March 2020 and was attributed to shifts in public behavior and hospitals avoiding elective care.^14^ However, emergency department admissions also decreased, and declines for admission for acute illness were also observed during this period, which may suggest less severe symptomatic patients generally stayed home or that some hospitalizations may have been coded with COVID-19 rather than AF as the primary diagnosis, further contributing to the observed reduction in primary AF hospitalizations.^15^ Consideration was also given to whether shifts in AF ablation billing from inpatient to observation status contributed to the observed trends. A supplemental analysis determined that ablation-related hospitalizations represented 5% to 8% of primary AF hospitalizations annually. A disproportionate decline in ablation-coded admissions from 2013 to 2014 (∼25% vs ∼5% for nonablation AF) was observed, consistent with the creation of the “Two-Midnight Rule” CMS policy which shifted short-stay ablation cases to observation status.^16^ Nevertheless, hospitalization trends after excluding ablation cases were largely preserved (Supplemental Figure 4), suggesting broader patterns in AF-related health care utilization were the predominant driver of the observed trend. The increase in admissions in recent years may also be partly attributable to the higher rates of detection of AF through consumer devices, with the incidence of newly diagnosed arrhythmia from smart devices ranging from 0.12% in a healthy population to 8% among hospitalized patients.^17^

Female sex conferred higher odds of readmission and mortality, likely reflecting greater thromboembolic risk, although whether this is concordant with sex-specific stroke admission trends warrants further investigation.^18^ In regards to socioeconomic factors, patients from higher income quartiles had lower adjusted odds of readmission and mortality on index admission compared to the lowest income quartile, similar to previous analysis of the NRD as well as in the context of other CV disease states.^19^, ^20^, ^21^ In addition, Medicare and Medicaid beneficiaries were found to have higher odds of readmission and mortality when compared to private insurance, mirroring previously identified findings which have been attributed to the higher comorbidities in these groups.^22^ Previous studies have also shown that government-provided insurance through Medicare or Medicaid are markers of clinical risk but also of poorer access to primary care relative to those with private insurance.^23^, ^24^, ^25^

The increasing number of Medicare beneficiaries throughout the study period also parallels the aging population of the primary AF population and mirrors previously reported expansions, which may contribute to poorer outcomes in regards to mortality and readmission.^26^ Given the strong age-linked relationship between Medicare enrollment and older age, insurance status in these models should be interpreted as reflecting residual risk beyond age, encompassing differential access to ambulatory follow-up and comorbidity burden not fully captured by age alone.

Overall, both populations of patients who experienced 30-day readmissions and index admission mortality were found to have higher rates of several comorbidities when compared to their counterparts who did not, including HF, diabetes, COPD, and renal disease. In addition, the proportions of these comorbidities increased in the population over the study period. Renal disease in particular demonstrated a strong association with readmission and mortality on index admission with adjusted ORs of 1.39 and 3.98 respectively, similar to previous studies.^27^ Patients with chronic kidney disease and AF are less likely to receive optimal anticoagulation and rhythm control strategies, partly due to concerns about drug safety and efficacy in renal impairment and this undertreatment may further increase the risk of adverse outcomes.^28^ In addition, renal disease amplifies the prothrombotic state seen in AF, increasing the risk of stroke and systemic embolism.^29^ At the same time, chronic kidney disease impairs platelet function and increases bleeding risk, complicating anticoagulation management and contributing to higher mortality.^30^ Shifting anticoagulation strategies in the study period may also play a role, as warfarin was the predominant choice of anticoagulation in 2010 before being supplanted by direct oral anticoagulants such as apixaban by 2017.^31^^,^^32^

COPD emerged as the strongest comorbidity predictor of 30 day readmission with adjusted OR of 1.48, reflective of the interconnected pathophysiological mechanisms of both conditions and bidirectional relationship in regards to mortality and readmission.^22^ HF also demonstrated a strong association with 30 day readmission and mortality with adjusted odds ratios of 1.38 and 1.83 respectively. This finding is similar to other studies, the ORBIT-AF registry demonstrated that patients with HF had higher hospitalization rates (HR: 1.31; 95% CI: 1.23-1.39) compared to AF patients without HF.^33^ In regards to mortality, a recent population-based study found median overall survival of only 3.15 years (95% CI: 3.08-3.21) after diagnosis of both conditions, with 1-year mortality of 31.8% and 10-year mortality of 80.2%.^34^

Interestingly, obesity was found to be protective for readmission and mortality despite adjustment for comorbidities. Multiple studies have described an inverse relation between obesity and outcomes in CV disease and may reflect a healthy coding bias for patients that have documented obesity codes.^35^, ^36^, ^37^ Unadjusted analyses showed higher crude mortality among patients with HTN and CAD, but these associations reversed after adjustment, suggesting confounding by age, comorbidity burden, and treatment factors (Supplemental Tables 1 and 2).

### Study Limitations

There are several limitations to our study. Unique patients are not identified across different years in the NRD, which prevents tracking 30-day readmission occurring in the month of December. The NRD is derived from hospital billing data and granular clinical data such as vitals, laboratory values, and cardiac testing results are not available. AF subtype analysis was not feasible across the full study period due to successive coding changes, as ICD-9-CM classified all AF under a single code through 2015, ICD-10-CM introduced broad subtype categories from 2016 to 2018, and granular subtype differentiation was not available until the 2019 ICD-10-CM restructuring. The last available year for the Healthcare Cost and Utilization Project NRD is 2022, and as such does not capture the postapproval era of pulsed field ablation, which received Food and Drug Administration approval in 2024 and may meaningfully alter hospitalization patterns, procedural volumes, and rhythm control utilization in ways that cannot yet be assessed using currently available administrative data. The multivariable models were fitted on pooled data across the study period; as such, the reported ORs reflect average associations over 2010 to 2022 and may not capture temporal variation in effect estimates. In addition, this analysis did not employ a competing risks framework; patients who died during index hospitalization were classified as nonreadmitted, which may bias estimates for factors independently associated with both outcomes, such as obesity and HLD. The NRD does not code some demographic data such race and ethnicity. The NRD does not provide information regarding mortality that occurs outside of the hospital setting.

## Conclusions

In this national analysis of AF hospitalizations, unique patient hospitalizations and readmissions experienced a modest overall net increase from 2010 to 2022, but had a notable decline in hospitalizations from 2014 to 2020, before ultimately rebounding in 2021 to 2022 following the acute phase of the COVID-19 pandemic. Patients with higher comorbidity and lower socioeconomic status experienced greater risk of readmission and mortality after an initial hospitalization. Identifying AF earlier and developing interventions to reduce the burden of AF are of value to reduce acute care hospitalization needs.

Perspectives **COMPETENCY IN MEDICAL KNOWLEDGE AND PATIENT CARE:** Clinicians should recognize that AF patients with renal disease, HF, COPD, and public insurance coverage are at substantially elevated risk for 30-day readmission and in-hospital mortality, and should prioritize targeted postdischarge follow-up and optimization of comorbidity management in these high-risk groups.

**TRANSLATIONAL OUTLOOK:** Future studies should examine the impact of contemporary rhythm control strategies including pulsed field ablation on national AF hospitalization trends, and develop targeted interventions to reduce readmission and mortality disparities among socioeconomically vulnerable and high-comorbidity AF patients.

## Funding support and author disclosures

The authors have reported that they have no relationships relevant to the contents of this paper to disclose.
