# Supplementary material for: National Trends in Atrial Fibrillation Hospitalizations, Readmissions, and Mortality, 2010 to 2022
Source: JACC Adv. 2026 Aug 12;5(9):103118. doi: 10.1016/j.jacadv.2026.103118 (PMC13482214; doi:10.1016/j.jacadv.2026.103118)
Supplement: Supplementary file 1 [file mmc1.pdf]

Supplemental 1. Unadjusted OR for 30-Day All-Cause Readmission Following Primary Atrial Fibrillation Hospitalization

Variable	Unadjusted Odds Ratio (95% CI)	p-value
**Age**		
**Age <65**	Reference	Reference
**Age 65–75**	0.98 (0.97–0.99)	<0.001
**Age >75**	1.33 (1.32–1.34)	<0.001
**Sex**		
**Male**	Reference	Reference
**Female**	1.16 (1.15–1.17)	<0.001
**Payer Status**		
**Private**	Reference	Reference
**Medicare**	1.55 (1.54–1.57)	<0.001
**Medicaid**	1.21 (1.19–1.23)	<0.001
**Self-Pay**	0.65 (0.63–0.67)	<0.001
**No Charge**	0.73 (0.67–0.79)	<0.001
**Other**	0.81 (0.79–0.83)	<0.001
**Median Income Quartile**		
**1st**	Reference	Reference
**2nd**	1.01 (1.00–1.02)	0.023
**3rd**	0.96 (0.95–0.97)	<0.001
**4th**	0.89 (0.88–0.90)	<0.001
**Comorbidities**		
**Obesity**	0.88 (0.87–0.89)	<0.001
**Heart Failure**	1.68 (1.67–1.69)	<0.001
**History of MI**	1.56 (1.53–1.59)	<0.001
**Hypertension**	1.22 (1.20–1.23)	<0.001
**Diabetes Mellitus**	1.32 (1.31–1.33)	<0.001
**Hyperlipidemia**	1.03 (1.03–1.04)	<0.001
**Coronary Artery Disease**	1.45 (1.44–1.46)	<0.001
**Cerebrovascular Disease**	1.35 (1.33–1.37)	<0.001
**Chronic Obstructive Pulmonary Disease**	1.72 (1.71–1.74)	<0.001
**Renal Disease**	1.70 (1.69–1.72)	<0.001

Supplemental 2. Unadjusted OR for In-Hospital Mortality During Index Admission for Primary Atrial Fibrillation Hospitalization

Variable	Unadjusted Odds Ratio (95% CI)	p-value
**Age**		
**Age <65**	Reference	Reference
**Age 65–75**	0.68 (0.65–0.70)	<0.001
**Age >75**	2.76 (2.68–2.84)	<0.001
**Sex**		
**Male**	Reference	Reference
**Female**	1.26 (1.22–1.29)	<0.001
**Payer status**		
**Private**	Reference	Reference
**Medicare**	2.35 (2.26–2.44)	<0.001
**Medicaid**	0.76 (0.71–0.81)	<0.001
**Self-Pay**	0.50 (0.44–0.57)	<0.001
**No Charge**	0.31 (0.21–0.45)	<0.001
**Other**	1.05 (0.95–1.16)	0.330
**Median Income Quartile**		
**1st**	Reference	Reference
**2nd**	1.02 (0.99–1.06)	0.135
**3rd**	0.91 (0.88–0.94)	<0.001
**4th**	0.82 (0.79–0.85)	<0.001
**Comorbidities**		
**Obesity**	0.51 (0.49–0.54)	<0.001
**Heart Failure**	2.78 (2.70–2.87)	<0.001
**History of MI**	4.94 (4.73–5.15)	<0.001
**Hypertension**	0.81 (0.78–0.83)	<0.001
**Diabetes Mellitus**	1.07 (1.03–1.10)	<0.001
**Hyperlipidemia**	0.62 (0.60–0.64)	<0.001
**Coronary Artery Disease**	1.23 (1.19–1.26)	<0.001
**Cerebrovascular Disease**	2.64 (2.52–2.76)	<0.001
**Chronic Obstructive Pulmonary Disease**	2.11 (2.04–2.17)	<0.001
**Renal Disease**	5.01 (4.86–5.16)	<0.001

Supplemental 3: Trends of demographics and comorbidities for primary AF hospitalization

Characteristic	Year, %													
	2010	2011	2012	2013	2014	2015	2016	2017	2018	2019	2020	2021	2022	P-Trend
Total No.	478,809	478,515	492,752	481,930	451,821	475,005	471,565	479,500	483,286	499,739	413,665	457,417	477,042	
Age, mean, y	69.9	70.1	70.0	70.1	70.2	70.5	70.4	70.6	70.6	70.9	70.8	71.2	71.7	<0.001
Female	50.5%	50.8%	50.0%	49.7%	50.0%	49.9%	49.5%	49.4%	48.9%	48.6%	47.7%	47.8%	47.8%	<0.001
Payer status														
Medicare	66.5%	67.0%	67.7%	68.4%	68.2%	69.1%	69.3%	70.0%	70.0%	70.5%	70.1%	71.0%	72.6%	<0.001
Medicaid	4.2%	4.3%	4.4%	4.5%	5.7%	5.7%	5.8%	5.9%	5.8%	5.7%	6.3%	6.3%	5.9%	<0.001
Private	23.9%	23.1%	22.0%	21.2%	21.0%	20.5%	20.4%	19.7%	19.7%	19.2%	18.7%	18.1%	17.1%	<0.001
Self-pay	2.7%	2.8%	2.9%	3.0%	2.5%	2.2%	2.1%	2.1%	2.3%	2.2%	2.2%	2.0%	1.6%	<0.001
No-charge	0.3%	0.4%	0.4%	0.4%	0.4%	0.4%	0.3%	0.3%	0.3%	0.3%	0.2%	0.2%	0.1%	0.001
Other	2.3%	2.5%	2.6%	2.5%	2.1%	2.2%	2.2%	2.0%	2.0%	2.0%	2.4%	2.5%	2.6%	0.736
Median household income quartile														
1st	28.2%	28.6%	29.2%	26.2%	26.0%	28.0%	27.8%	27.9%	26.8%	27.6%	27.4%	26.6%	26.5%	0.093
2nd	25.3%	24.4%	25.2%	27.6%	27.8%	25.9%	26.7%	28.6%	29.1%	27.4%	29.9%	27.9%	28.0%	0.003
3rd	23.6%	25.0%	23.3%	24.8%	24.0%	25.6%	25.6%	24.7%	25.1%	25.4%	23.8%	24.7%	25.2%	0.187
4th	22.9%	22.0%	22.3%	21.4%	22.3%	20.5%	19.9%	18.8%	19.0%	19.5%	18.9%	20.8%	20.3%	0.004
Comorbidities														
Obesity	12.3%	13.2%	14.6%	16.0%	17.4%	18.9%	20.3%	21.6%	22.5%	23.0%	24.6%	25.4%	25.3%	<0.001
Heart failure	28.5%	29.1%	29.2%	31.4%	34.0%	35.9%	36.8%	38.2%	40.2%	41.3%	43.7%	44.0%	42.9%	<0.001
History of myocardial infarction	1.4%	1.5%	1.6%	1.7%	1.8%	2.0%	2.0%	2.6%	3.2%	3.7%	5.1%	5.6%	6.0%	<0.001
Hypertension	70.1%	71.1%	72.2%	73.0%	74.2%	75.8%	77.2%	79.0%	79.8%	80.5%	81.1%	81.5%	81.8%	<0.001
Diabetes	26.6%	26.8%	27.6%	28.3%	29.4%	29.4%	27.8%	28.3%	28.5%	28.7%	28.8%	29.1%	28.9%	0.011
Lipid disorders	44.1%	45.0%	46.5%	47.6%	48.4%	49.3%	49.9%	51.4%	52.2%	54.2%	55.6%	57.3%	58.4%	<0.001
CAD	33.3%	33.2%	33.4%	33.7%	34.5%	34.7%	35.3%	35.6%	36.4%	37.3%	38.3%	38.4%	38.7%	<0.001
CVD	4.6%	4.5%	4.5%	4.6%	4.8%	4.5%	4.0%	4.1%	4.1%	4.3%	4.5%	4.5%	4.5%	0.295
COPD	15.5%	16.0%	16.3%	16.8%	17.0%	17.8%	19.4%	19.7%	19.5%	19.9%	19.6%	18.7%	18.5%	<0.001
Renal disease	15.4%	16.5%	17.5%	20.1%	22.1%	23.5%	24.3%	24.5%	25.5%	27.2%	28.7%	28.9%	29.1%	<0.001

Supplemental 4: Trends of Primary AF Hospitalizations With and Without Ablation during index hospitalization

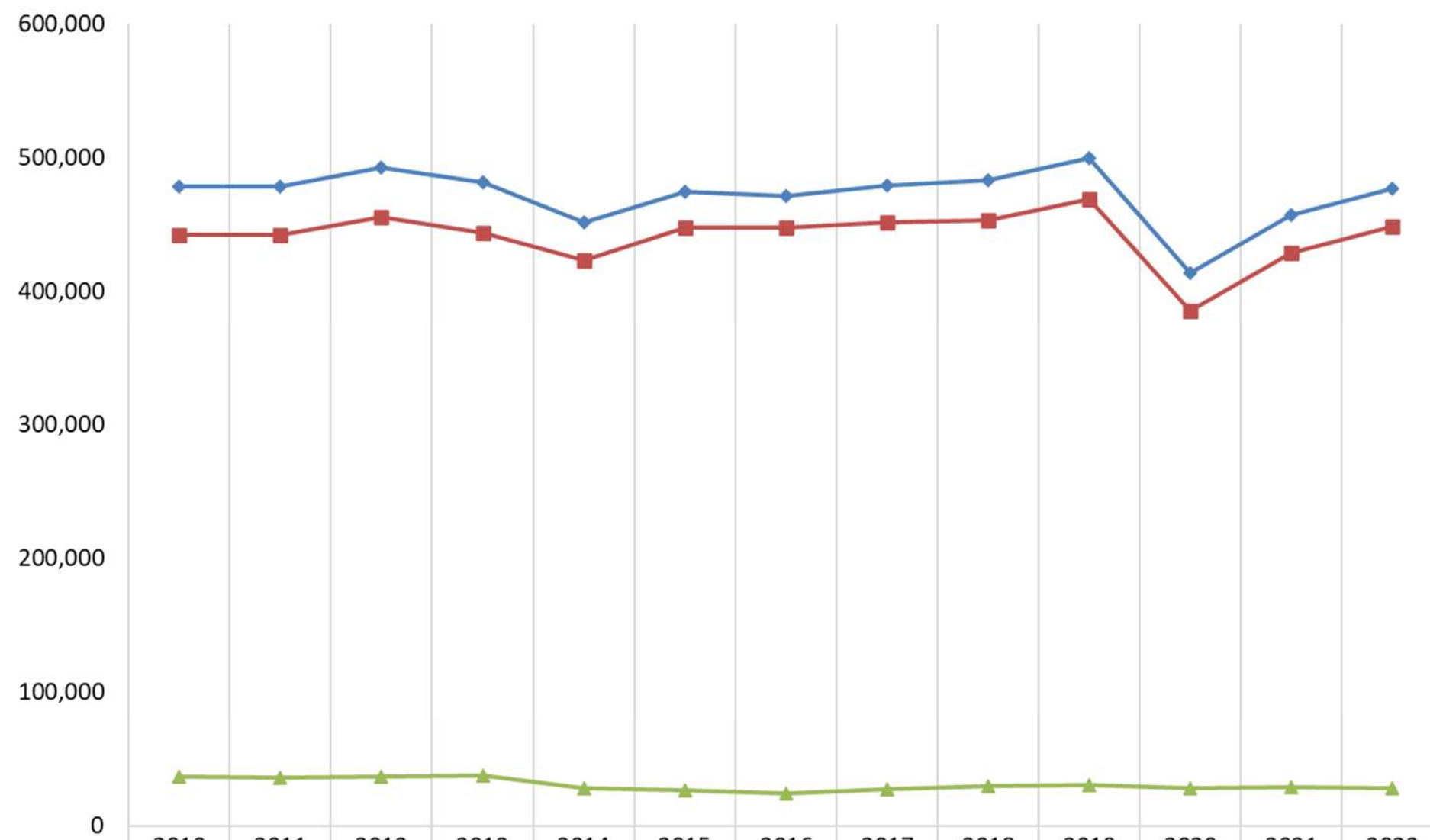

	2010	2011	2012	2013	2014	2015	2016	2017	2018	2019	2020	2021	2022
Primary AF hospitalizations	478,809	478,515	492,752	481,930	451,821	475,005	471,565	479,500	483,286	499,739	413,665	457,417	477,042
Primary AF without ablation performed during hospitalization	442,109	442,707	455,720	443,927	423,249	448,278	447,576	451,851	453,520	468,924	385,473	428,718	448,481
Primary AF with ablation performed during hospitalization	36,700	35,809	37,032	38,003	28,572	26,726	23,989	27,648	29,766	30,815	28,191	28,699	28,562

Supplemental 5: STROBE Statement—checklist of items that should be included in reports of observational studies

	Item No.	Recommendation	Page No.	Relevant text from manuscript
**Title and abstract**	1	(*a*) Indicate the study's design with a commonly used term in the title or the abstract	1	Serial cross-sectional study; stated in title and Methods
		(*b*) Provide in the abstract an informative and balanced summary of what was done and what was found	1-2	Structured abstract with all 5 required sections
**Introduction**				
Background/rationale	2	Explain the scientific background and rationale for the investigation being reported	1	AF is most common arrhythmia; limited contemporary longitudinal data
Objectives	3	State specific objectives, including any prespecified hypotheses	1	To identify national trends in AF hospitalizations, readmissions, and mortality 2010-2022
**Methods**				
Study design	4	Present key elements of study design early in the paper	4	Serial cross-sectional analysis using NRD 2010-2022
Setting	5	Describe the setting, locations, and relevant dates, including periods of recruitment, exposure, follow-up, and data collection	4	NRD all-payer US inpatient database; January 2010 to November 2022
Participants	6	(*a*) *Cohort study*—Give the eligibility criteria, and the sources and methods of selection of participants. Describe methods of follow-up<br>*Case-control study*—Give the eligibility criteria, and the sources and methods of case ascertainment and control selection. Give the rationale for the choice of cases and controls<br>*Cross-sectional study*—Give the eligibility criteria, and the sources and methods of selection of participants	4	Adults ≥18 with primary/secondary AF; ICD-9-CM 427.31, ICD-10-CM I48.1-I48.91; atrial flutter and December discharges excluded
		(*b*) *Cohort study*—For matched studies, give matching criteria and number of exposed and unexposed<br>*Case-control study*—For matched studies, give matching criteria and the number of controls per		

		case		
Variables	7	Clearly define all outcomes, exposures, predictors, potential confounders, and effect modifiers. Give diagnostic criteria, if applicable	4-5	Primary AF hospitalization, 30-day readmission, in-hospital mortality defined; comorbidities via ICD and CCS code
Data sources/ measurement	8*	For each variable of interest, give sources of data and details of methods of assessment (measurement). Describe comparability of assessment methods if there is more than one group	4-5	NRD administrative claims; ICD-9/10 codes consistently applied
Bias	9	Describe any efforts to address potential sources of bias	4-5	Nationally representative all-payer database; survey weights applied
Study size	10	Explain how the study size was arrived at	4-5	All adult AF hospitalizations included; entire database population used

Continued on next page

Quantitative variables	11	Explain how quantitative variables were handled in the analyses. If applicable, describe which groupings were chosen and why	4-5	Age: <65, 65-75, >75; income: quartiles 1-4; payer status categorical
Statistical methods	12	(*a*) Describe all statistical methods, including those used to control for confounding	4-5	Survey-weighted logistic regression; clustered at hospital level; a priori variable selection
		(*b*) Describe any methods used to examine subgroups and interactions	N/A	No subgroup analyses performed
		(*c*) Explain how missing data were addressed	4-5	Missing data not imputed; race/ethnicity unavailable noted as limitation
		(*d*) *Cohort study*—If applicable, explain how loss to follow-up was addressed<br>*Case-control study*—If applicable, explain how matching of cases and controls was addressed<br>*Cross-sectional study*—If applicable, describe analytical methods taking account of sampling strategy	N/A	Not applicable; cross-sectional design
		(*e*) Describe any sensitivity analyses	8	Supplemental ablation sensitivity analysis (Supplemental Figure 4)
**Results**				
Participants	13*	(a) Report numbers of individuals at each stage of study—eg numbers potentially eligible, examined for eligibility, confirmed eligible, included in the study, completing follow-up, and analysed	5-6	56,104,334 total AF hospitalizations; 6,141,046 primary AF; 5,528,396 unique patients
		(b) Give reasons for non-participation at each stage	N/A	Administrative database; all eligible hospitalizations captured
		(c) Consider use of a flow diagram	N/A	Flow diagram not included; inclusion/exclusion in Methods text
Descriptive data	14*	(a) Give characteristics of study participants (eg demographic, clinical, social) and information on exposures and potential confounders	5-7	Demographics and comorbidities in Tables 1 and 3; trends in Figure 2
		(b) Indicate number of participants with missing data for each variable of interest	N/A	Race/ethnicity unavailable noted as limitation
		(c) *Cohort study*—Summarise follow-up time (eg, average and total amount)	N/A	Not applicable; cross-sectional design
Outcome data	15*	*Cohort study*—Report numbers of outcome events or summary measures over time		

		Case-control study—Report numbers in each exposure category, or summary measures of exposure		
		Cross-sectional study—Report numbers of outcome events or summary measures	5-7	892,254 30-day readmissions (16.1%); 53,084 deaths (0.96%); annual counts in Figure 1
Main results	16	(*a*) Give unadjusted estimates and, if applicable, confounder-adjusted estimates and their precision (eg, 95% confidence interval). Make clear which confounders were adjusted for and why they were included	5-7	Adjusted ORs with 95% CI in Tables 2 and 4; a priori variable selection
		(*b*) Report category boundaries when continuous variables were categorized	4-5	Age: <65, 65-75, >75; income: quartiles 1-4
		(*c*) If relevant, consider translating estimates of relative risk into absolute risk for a meaningful time period	N/A	Absolute risk translation not performed

Continued on next page

Other analyses	17	Report other analyses done—eg analyses of subgroups and interactions, and sensitivity analyses	8	Supplemental ablation analysis (Supplemental Figure 4)
**Discussion**				
Key results	18	Summarise key results with reference to study objectives	7-10	Net increase 2010-2022; decline 2014-2020; post-pandemic rebound; renal disease strongest predictor
Limitations	19	Discuss limitations of the study, taking into account sources of potential bias or imprecision. Discuss both direction and magnitude of any potential bias	10-11	December tracking; billing data; subtype coding; PFA era; pooled model; competing risks; race/ethnicity
Interpretation	20	Give a cautious overall interpretation of results considering objectives, limitations, multiplicity of analyses, results from similar studies, and other relevant evidence	7-10	Results interpreted cautiously; insurance ORs reflect residual risk beyond age
Generalisability	21	Discuss the generalisability (external validity) of the study results	10-11	NRD nationally representative all-payer database
**Other information**				
Funding	22	Give the source of funding and the role of the funders for the present study and, if applicable, for the original study on which the present article is based	1	No funding received. No conflicts of interest.

*Give information separately for cases and controls in case-control studies and, if applicable, for exposed and unexposed groups in cohort and cross-sectional studies.

Note: An Explanation and Elaboration article discusses each checklist item and gives methodological background and published examples of transparent reporting. The STROBE checklist is best used in conjunction with this article (freely available on the Web sites of PLoS Medicine at http://www.plosmedicine.org/, Annals of Internal Medicine at http://www.annals.org/, and Epidemiology at http://www.epidem.com/). Information on the STROBE Initiative is available at www.strobe-statement.org.
